# Recherehes Cliniques sur le Diagnostic, l'Etiologie, la Curabilité, &c. de la premiere Période de la Phthisie Pulmonaire

**Published:** 1842-07-01

**Authors:** 


### RECHERCHES CLINIQUES SUR LE DIAGNOSTIE, L'ETIOLOGIE, LA CURABILITÉ, &c., DE LA PREMIERE PÉRIODE DE LA PHTHISIE PULMONAIRE.

Clinical Researches on the Diagnosis, Etiology, Curability, &c. of the first Stage of Pulmonary Phthisis. By M. *Jules Fournet*, M.D. Paris.

[Second Part.]

IN a preceding number of this Journal we presented the reader with an analysis of the first part of this work. In the second part, of which we are now about to give an outline, the author applies the principles established in the first part to the detection of pulmonary phthisis in its first stage—in that stage, in fact, in which, according to the ordinary modes of investigation, not the slightest suspicion could be entertained of the presence of tubercular deposition. The second and third stages of phthisis were the only ones studied or known in Laennec's time; that accurate observer candidly acknowledged, that he was unable to detect the presence of small crude tubercles, how numerous soever they might be. The universal failure of the resources of art in the treatment of phthisis in its second and third stages, first induced our author to devote his energies and transfer all his efforts to establish a diagnosis of the disease in its first and earliest stage. He adduces the testimony of Andral with respect to the precision and exactness with which he was able to announce tubercular infiltration at its very commencement, at a stage in which the actual state of science would not enable an observer even to suspect the presence of tubercle. The principal object of this second part is then the exposition of the means of diagnosing the first stage of phthisis. These means, the author tells us, he sought even in the etiology of the disease, and fairly enough, in as much as the existence of the cause is a further reason for suspecting the presence of the effects. Thus he interrogates successively the circumstances of constitution, of hereditary predisposition, of age, sex, menstruation, pregnancy, previous diseases of the respiratory system, as well as of the other systems, habitation, diet, profession, season, climate, &c.; all for the purpose of ascertaining the degree of influence, remote or proximate, of these circumstances on the development of phthisis. After the signs which may be derived from the consideration of the causes, he then passes in review the signs which may be obtained from an examination of the respiratory and other systems. With respect to the first of these systems, he examines in succession the signs afforded by the stethoscope, percussion, aconophony, palpation, inspection, by the expectoration, the peculiar sensations of the patient, and the exact seat of the several physical signs. Next comes the analysis of the general phenomena; then the varieties sometimes presented by the different orders of facts just mentioned. After having considered these separately, for the purpose of more easily tracing their especial characters, he then examines their value, when combined. Next he considers the course of the first stage of phthisis, and its division into three phases or epochs, to each of which corresponds a certain number of signs. He then notes the relations existing between symptomatology and pathological anatomy, the numerous relations with respect to the moment when the first symptoms appeared, the course of the disease, the different forms which it may assume, and the degrees in which it presents itself; important relations, which, when we ascend to the first manifestation of the symptoms and the lesions, tend to shew what order of symptoms, local or general, appears first, and whether the disease is primarily local or general. After these several considerations, which make known the course of the affection during its first period, and which place the observer in an advantageous position with respect to treatment, comes the differential diagnosis of the first stage of phthisis. He concludes by laying down the general data on which the treatment of this first stage of the disease should be founded, and developing the erroneousness of the opinion, which holds as a principle the incurability of phthisis in its first degree, and the frequent cure of it in its third stage: a fatal error which tends to destroy the spirit of investigation, and deprives science and humanity even of the idea of opposing the evil, and causes the physician to allow the only moments to pass by when the combined influence of nature and art may be able to effect something, and reserves our hopes for a period, when death alone is to be expected. Such is the plan of this second part of our author's work, embracing nearly the entire history of the first stage of pulmonary phthisis.

In order to obtain signs from the constitution, with respect to the diagnosis of phthisis, he considers the several structures of the body; as the osseous, muscular, cutaneous, dental, ungual and nervous structures; we shall state the results.

The greater or less development of the chest does not seem to him as closely connected, as is generally supposed, with tuberculization of the lungs; he concludes that, out of a given number of phthisical patients, about one third have the chest well developed.

With respect to the muscular system, he finds in the list of his phthisical patients several athletic individuals; a much greater proportion than is generally supposed. Out of a given number nearly one third have the muscular system *ordinarily* developed.

When questioned with respect to their state of em-bon-point before the commencement of their illness, the greater number of his patients have stated, either that they were very fat, or at least in tolerably good condition. In about three fourths of his phthisical patients the skin was observed to be extremely fair, fine and delicate. With respect to the hair very few had it fair; the greater number had it of a brown colour, and some had it even of a jet black. He was particular in observing the nails and fingers of his patients; and by no means considers these parts as affording such diagnostic signs as they are supposed to do. He infers from his tables, that the fusiform or *Hippocratic* fingers, considered by some as so strikingly characteristic of phthisis, are more frequently found in persons not presenting the slightest trace of tubercular disease. The peculiar appearance of the teeth he considers of no value in diagnosing phthisis. The intellectual and sensorial faculties presented at least the ordinary rate of development. In a word, the weak constitution and lymphatic temperament are the states most frequently found by him in his phthisical patients; not however the extremely weak or extremely lymphatic temperament, but those of the common or ordinary form. The coincidence of a strong, muscular constitution, of a well-developed and well-formed skeleton, and of a sanguineous temperament, with pulmonary phthisis, is much more frequent than is generally supposed. The number of patients so conditioned form about one third of the entire; this result is somewhat different from what is generally admitted; it is probably owing to the circumstances under which his cases were taken; these circumstances were such that the number of cases of hereditary phthisis were considerably less than that of acquired or accidental phthisis, and it is well known that debility of constitution belongs much more to the first than to the second of these two forms of phthisis.

The number of cases of phthisis, independent of any predisposition derived from hereditary transmission and constitution, arising merely under the influence of certain anti-hygienic conditions is much greater, according to our author's experience than is generally believed. This fact concurs with the preceding in explaining the rather frequent coincidence of pulmonary phthisis with a strong, muscular constitution and a sanguineous temperament.

The conclusion which he draws from all this is, that the constitution of the patient, considered separately, is an almost valueless element in solving the problem of the diagnosis of the first stage of pulmonary phthisis, excepting, however, those cases where the suspicion of phthisis should happen to coincide with the more marked forms of the lymphatic temperament, cases rather rare in adults, but much more so in persons of a more advanced age. But if the consideration of the individual's constitution is connected with certain other circumstances favorable or unfavorable to the opinion of phthisis, it may acquire a confirmatory value, or one of a merely additive nature. Thus the coincidence of a strong constitution and a sanguineous temperament with the absence of the anti-hygienic conditions capable of developing phthisis should make us reject the already conceived suspicion of the existence of phthisis; whilst this isolated fact, (a strong constitution and a sanguine temperament) cannot decide the question, either affirmatively, or negatively. In children the consideration of the constitution is of much more value.

The author well remarks that all he has said on the constitution, is referrible to the primary characters of the constitution of the patients, and not to the changes which their constitution may have undergone in consequence of the development of their phthisis.

*On Hereditary Transmission.*—According to our author, and indeed according to the experience of all pathologists, both ancient and modern, the hereditary transmission of phthisis cannot be called in question. Our author has studied the subject of hereditary transmission with respect to the father, mother, brothers, and sisters of his patients. The principal results of his investigations have been, that the absence of phthisis in the immediate relatives of a patient is not a sufficient reason for excluding the idea of it in the patient himself. Patients he often observed to die phthisical, who, when subjected to a strict examination with respect to the health of their parents and friends, always denied the existence of any symptoms of phthisis in them; their parents, in fact, were still living and in good health, or if they were dead, they died of some disease altogether different from phthisis. The patients in question had become phthisical under the influence of certain anti-hygienic circumstances, personally confined to themselves. Now if a well conducted examination ascertained the absence of phthisis, past or present, in the father and mother of any given patient, and the absence in this patient of the anti-hygienic circumstances just mentioned, there would exist the strongest grounds for rejecting the suspicion of phthisis; yet, under certain circumstances, very rare to be sure, phthisis is observed to become developed, independently of all influence of hereditary transmission, or indeed of any appreciable cause.

For the reasons above assigned, if there existed no probability of phthisis in the father and mother, and if a brother or sister died phthisical under the influence of anti-hygienic circumstances, such a precedent should not at all influence the judgment of the physician on the patient in question, inasmuch as the influence of these circumstances was entirely personal.

On this part of the subject a question may be raised; namely, whether, cæteris paribus, acquired, or, as we may call it, accidental phthisis, does not attack certain constitutions in preference to others, or even exclusively; secondly, whether, in case of an affirmative answer being given to the preceding question, there is a sufficient similitude in the organization of children of the same parents to warrant the physician in apprehending or dreading in one of them the phthisis which has developed itself in the other under the influence of anti-hygienic circumstances. The conclusions to which our author's experience has led him, on this question is, that—1st, it does not appear probable that accidental phthisis requires for its development certain constitutional conditions—2nd, that it is possible that the anti-hygienic circumstances capable of developing phthisis act more effectively and more rapidly on certain constitutions than on others; but, as a general rule, there is not sufficient uniformity in the organization of infants of the same parents, to enable one to conclude, the same anti-hygienic circumstances being given, either that they will produce, or that they have already produced in the one the same effects as they had produced in the other. Certain professions are considered as predisposing to phthisis; in countries where these professions are in vogue, several members of one and the same family are employed at the same kind of work; now, in such cases, some are observed to become phthisical, others not so. In fact, the conclusion which our author draws from his experience on this matter is; that any phthisis, developed in a family under the influence of anti-hygienic circumstances, setting aside altogether the influence of hereditary taint, remains a personal fact, confined to the individual so affected, and that no consequence can be drawn from it for the other children of the same family.

Acquired phthisis may become developed even at an advanced period of life. It recognizes in this respect no other law save the anti-hygienic conditions, which develop it. A father, born of healthy parents, his children, now full grown; he experiences reverses of fortune, and in order to satisfy the wants of his family and the education of his children, he is forced suddenly to change the tranquil and easy life which he hitherto enjoyed, for a life of privations, anxiety and annoyances. His health, heretofore good, becomes changed from day to day, and in a little time he dies of acute phthisis. At a post-mortem his lungs are found pervaded with miliary tubercles. Now, under such circumstances, the health of the father does not at all compromise that of the children, and consequently no comparison can be instituted between the nature of the disease which has carried off the father and that of the disease now affecting one of the children.

The vast importance of the question regarding the hereditary transmissibility of phthisis makes us dwell longer on this portion of our author's work than we otherwise should do. His remarks on this subject are very pertinent, and very practical. He well remarks that children may have been *conceived* subsequently to the manifestation of an organic process of *acquired phthisis* in one of the parents. What will be the result, he asks, of this in comparison with the influence of predispositions to hereditary phthisis? The conclusion to which his experience brings him, is; that the hereditary influence, to which children are subjected who are born of tubercular parents, is less when the phthisis of which the parents have died, was acquired, than when it was a family inheritance for themselves. Phthisis, then, in its first generation should be less dangerous in its hereditary transmission, than phthisis in its second generation.

Being given a family consisting of several children, the one of the two heads of which, or even both, have died of phthisis, the hereditary influence which the children receive, may manifest itself in several different ways, and such manifestation appears not to be subjected to any established law. This influence sometimes appears to exhaust itself on some one of the children; but when the family is large, such influence is seen to extend to several of its members. It generally happens that some members of the family are exempted from the baneful influence which seems to exhaust itself on the others, and they are observed to enjoy very good health. It rarely happens that all the children born of a consumptive father or mother die of phthisis. But as it is impossible to judge at what limit the effects of the tubercular predisposition will stop, and what will be the number of its victims, the physician should always entertain the greatest apprehensions regarding a patient, which excites suspicions of pulmonary phthisis, and who has lost a father or mother of this disease, or some of his brothers or sisters.

With respect to the influence of age in the production of phthisis, our author remarks that the disease may be developed at all ages; we have sufficient proof to satisfy us that it may become developed even in old age. Our author states that he has frequently found tubercles in the lungs of old persons, 60 or 70 years of age; these tubercles were crude in some and softened in others. The age however cannot furnish us with any sure aid in diagnosing the presence of phthisis.

With respect to the assistance to be derived from the manner in which the uterine functions are performed in the diagnosis of phthisis in its first stage, our author states that it is impossible to derive any useful data for this diagnosis from such a source; and for this he assigns a very good reason: namely, that the derangements of menstruation which generally accompany phthisis, are an effect and not a cause of the organic process which is going on in the lungs and in the rest of the system; that this process does not re-act on the uterine functions, until a period when the local and general sufficiently establish the diagnosis; lastly, that these derangements of the uterine functions are subject to too much irregularity in their appearance, and may be produced by too many different causes, to allow any one to establish any precise connexion between them and the process of pulmonary tuberculization.

Our author next considers the diseases from which the patient suffered as diagnostic signs; and first, the diseases unconnected with the respiratory system. He comes to the conclusion that the diseases of the cutaneous and mucous systems are those which appear most frequently in phthisical cases, and to be most immediately connected with tuberculization of the lungs, and the general tubercular cachexy. He next considers how far diseases of the respiratory system influence the development of phthisis; and he commences with bronchitis. The conclusion to which he comes on its influence, a conclusion perfectly in accordance with the opinions of Andral and Louis, is, that in some patients the catarrhal affections observed are effects of the tubercular cachectic state, or of tuberculization already existing; and that in others they are the exciting cause of phthisis to which their constitution and hereditary circumstances predispose them; they are sometimes the signal that the organic process which had hitherto lain dormant, or had been insidious and imperceptible in its course, has now commenced. With respect to the influence of pneumonia in producing tubercular disease, his opinion is that it is an affection wholly independent of tuberculization of the lungs, and that it is not related to it either as an exciting cause, or as an effect of the irritation produced by the presence of tubercles in the lungs; so that the consideration of its previous existence or non-existence can be of no use in the diagnosis of the first stage of pulmonary phthisis. The same thing he thinks cannot be said of pleuritis, which under certain circumstances may act as a determining cause of phthisis. The conclusions to which his experience leads him, are:—1st, that the passing of pleuritis into the chronic state should not necessarily make us suppose the existence of tubercular deposition on the side where it occurs;—2nd, that from it, if this fact happens to an individual, who evinces no signs whatever of a predisposition to pulmonary phthisis, and who has been suddenly surprized, in the midst of ordinary good health, by an attack of pleurisy which has come on under the influence of causes more or less appreciable, and which has passed into the chronic state in consequence of the imprudence of the patient, there may be a certainty that no tubercles pre-existed. 3d, But that if this state continues, and lasts for a considerable time, and if the patient's health evinces a tendency to become deteriorated, and if besides this we ascertain by the ordinary signs the existence of a false membrane around the lungs, there will be good reason to fear or even to suspect tubercular deposition in such false membrane as well as in the subjacent lung; 4, whilst, we may have an almost absolute certainty of it, if we observe the strength gradually to diminish, and that some general symptoms of hectic fever come on without any other cause capable of accounting for them. With the exception of the case just mentioned our author conceives that pleuritis, like bronchitis, may, according to circumstances, become an exciting cause of tubercular deposition to which the patient is already predisposed, or be only the effect of tuberculization already existing. In the latter case we generally see it appear without any known cause, then disappear and return within a very short space of time. With respect to hæmoptysis our author has not observed it as frequently as the accounts of some authors would lead one to suppose. This however may probably be owing to this circumstance, viz. that a considerable number of his cases bear only on the first stage of phthisis, a period of the disease at which the hæmoptysis which serves to mark its progress has not yet appeared. Our author concludes that the number of patients in whom hæmoptysis may enter as an element in the diagnosis of the first stage of phthisis is much more limited than one would be led to believe from the accounts of some authors, and as it is far from being easy to distinguish, within the limits of the first stage of phthisis, whether the hæmoptysis is connected with the existence of tubercles in the lungs, or with other morbid states, such as diseases of the heart, &c. it follows that the importance of hæmoptysis as a means of diagnosing the first stage of phthisis is proportionally diminished. Yet as the attacks of hæmoptysis connected with the existence of tubercles are infinitely more numerous than those depending on any other cause, and as certain circumstances may assist in distinguishing to what morbid conditions they belong, we may attach considerable importance to the consideration of this element of diagnosis.

We next come to the consideration of active sanguineous congestion of the lungs, and of hæmoptysis, as causes of tuberculization. In order to determine in what sense and within what limits these are connected with the ætiological history of phthisis, he lays it down as an established fact, that neither the one or the other has the power of producing this disease, but so far as there exists a predisposition, either congenital or acquired, to tuberculous formation; that their action is confined merely to calling this predisposition into play, and to directing its development to the organs in which they themselves are seated, and to rendering more or less rapid, and more or less intense, the morbid movements connected with the tuberculous cachexy. Clinical facts prove that hæmoptysical attacks, considered as signs of the first stage of phthisis, assume a peculiar character of severity, when they coincide with a delicate frame, a lymphatic temperament, a narrow chest, pale and flabby flesh, a certain flush of the cheeks, and with the circumstance of parents who died phthisical; because, in these cases, they are most commonly or almost always, connected with the existence of phthisis.

This influence of pulmonary congestion and hæmorrhage being acknowledged, may be accounted for in two ways. On the one hand, it may be supposed that they both have the effect—1st, of directing to the lung those morbid movements which the tuberculous cachexy brings with it, from which movements the process of tuberculization and the deposition of tuberculous matter in the substance of the lung result; 2nd, or else of provoking new tubercular formations, and of accelerating the progress of those already existing, when the localization of the morbid process is already effected. On the other hand, in cases of hæmoptysis, whether tubercles already exist, or not, it may be supposed that the extravasated blood, on condition that a predisposition to tuberculous formations exists, furnishes itself the materials of tubercle, as happens in the case of different other morbid productions. In the first case, the predisposition to tubercles would only have been evoked, localized, and excited in its effects; in the second case, the hæmorrhage would have concurred more directly in producing these effects by furnishing the materials. Our author considers at great length the comparative value of these two opinions, and concludes by stating, that active sanguineous congestions of the lungs and hæmoptysis, provided a predisposition to tuberculous formations exists, have a tendency to direct to and concentrate on the lungs the deposition of tubercle; and when it is once established, they have a tendency to perpetuate and accelerate it.

Our author next considers the diagnostic signs derivable from the antihygienic conditions in which the patient may be placed. As there is nothing very novel, however, in this part of his work, we shall pass on to the diagnostic signs furnished by the examination of the respiratory system. And first for the auscultation of the respiration.

1. *The Modifications produced in the Inspiratory Murmur.*—In the first period of pulmonary phthisis, these modifications of the inspiratory murmur are:—an increase of intensity, by which this murmur rises from 10

to 12, 15, or 18.* This increase is rather constant. It increases in a ratio directly proportional to the physical alteration of the organ. Next a diminution in the duration, occurring simultaneously with the increase in the intensity, but which is less constant and less marked. It depresses the inspiratory murmur from 10 to 8, 7, or even to 6 or 5, but very rarely below this. It sometimes happens that the intensity and duration of the inspiratory murmur do not undergo any appreciable change: in this case it retains its normal representative number, and the expiratory murmur is almost the only one changed.

The same, however, cannot be said of the changes produced in the soft, dry, humid character; in fact, in the first stage of pulmonary phthisis, the inspiratory murmur is observed to be dry, hard, rough and as it were difficult to be produced. The impression which the ear receives from it is quite peculiar. If we auscult the anterior part of the chest from below upwards in a patient labouring under the commencement of phthisis in the apex of the lungs, we shall be struck, we cannot fail to be struck, with the successive transition from the soft, easy, mellow character of the inspiratory murmur to the character of roughness and dryness, which it presents towards the summits. This character increases perceptibly, according as the tuberclar infiltration increases. It can no longer be perceived as soon as the changes of quality have attacked the inspiratory murmur.

2. *Modification in the Expiratory Murmur.*—The expiratory murmur uniformly presents, in the first stage of phthisis, an increase both of intensity and duration, an increase which may raise it from 2 to 20. This increase takes place by successive gradations to 4, 6, 8, 10, &c. The numbers representing it are, in general, a tolerably exact measure of the progress of the disease. The law which regulates the increase in the duration and intensity of the expiratory murmur is, according to our author, very constant, such increase not being subject, like certain other modifications of the normal sounds, to occasional disappearance; it is continued and regular in its ascending course; and affects uniformly and simultaneously both the duration and intensity of the expiratory murmur; sometimes, however, it affects the intensity but little, but it affects the duration much more. This circumstance might lead one into error, if he were not well practised in distinguishing in the murmur under examination the duration from the intensity. In the exceptional case just alluded to, the expiratory murmur remains weak enough; but the ear easily distinguishes that it is more prolonged than in the normal state. Even when the bronchial character has come to attach itself to the expiratory murmur, it may still be shewn that the new murmur resulting from it is more developed than if it had been affected by an alteration in its quality, without a previous increase in its intensity and duration. It must not, however, be forgotten that these changes of quality, when they affect any

* In the first part of this work, the representative number of the healthy inspiratory murmur was stated to be 10, that of the healthy expiratory murmur being two.—*(Rev.)*

murmur whatever, have the effect of making it appear a little increased in intensity and duration, that thenceforward the appreciation of these two characters should, in order to be exact, be made before the time when the sounds of inspiration or expiration are attacked by changes of quality.

The character of roughness, difficulty, and of dryness, belong to the expiratory as well as to the inspiratory murmur. It is, in general, so much the more perceptible according as the increase of the expiratory murmur is more marked. It becomes inappreciable, when the changes of quality have come to affect this murmur. The changes of quality are of very great importance in the diagnosis of the first stage of phthisis. Though appearing at a somewhat later period than the preceding signs, they manifest themselves with sufficient frequency to warrant the observer in being influenced in his treatment by the diagnosis they furnish. These changes are regulated by constant laws, applicable to all patients and all forms of phthisis. They are sufficiently clear and distinct to be easily appreciated by any person conversant with the practice of auscultation.

The alteration of *quality* in the respiratory murmurs, setting out from the time of their first appearance to the time when they have attained their maximum of intensity, pass through successive gradations which express accurately the increasing progress of the disease. They consist at first merely in a souffle a little *clearer* than the natural souffle of inspiration or expiration ; they pass from this to the *resonant, blowing,* and then the bronchial quality. This bronchial first exists in a *first* degree, then passes on to the *second* and *third* degrees, and ultimately assumes the *cavernous* or *amphoric* character, according to the extent of the cavity formed within the pulmonary tissue. Such are the successive gradations which the quality of the inspiratory and expiratory murmurs may pass through in the course of phthisis. When they have attained the bronchial character, these changes of quality still belong to the first stage of phthisis ; beyond this, they belong to the subsequent stages of the disease. However we must not infer the non-existence of cavities from the absence of the cavernous and amphoric characters ; for it sometimes happens that, in certain anatomical conditions of the diseased structures, the bronchial character in the second or third degree, which exists in a part close to the ear, obscures or altogether effaces a slight degree of cavernous character seated in the central portions of the lung.

The modifications occurring in the quality of the respiratory sounds have the uniformly constant character of appearing first in the expiratory murmur, and of not extending to the inspiratory murmur till a later period. Thus, during a certain portion of the first stage of pulmonary phthisis, the expiration alone presents changes of quality ; it is not till a subsequent period that the same modification appears in the inspiration. Thus we have, in this part, a sure means of judging the progress of the disease. We may even go farther in this judgment regarding the progress of the disease : by a law just as constant as the preceding, the changes of quality retain in the expiration a greater development than in the inspiration, as long as we are in the first degrees of the scale of gradations : then they lose successively this pre-eminence in the expiration, according as the last degrees of this scale become developed. Thus the clear souffle, the resonant and blowing qualities, and the bronchial character in its different degrees, always appear more marked in the expiration than in the inspiration. Thus we already hear the blowing quality and the bronchial character during the first of these two murmurs, at the time that the clear souffle and the resonant quality have scarcely appeared in the second; whilst, according as the last stages of pulmonary phthisis become developed, we find the inspiratory and expiratory murmurs affected more and more equally, by the cavernous and amphoric characters.

Taking a rapid survey of the modifications produced in the respiratory murmurs in the first stage of pulmonary phthisis, we see that the modifications of the expiratory murmur are much more important for diagnosis than those of the inspiratory murmur, and above all they possess the advantage of being referred to a much earlier period of the disease. The modifications in the duration and intensity of the respiratory murmurs appertain to a period of phthisis antecedent to the time when the changes in the soft, easy, mellow character, and the alterations of quality are observed; the alterations in the duration and intensity of the expiration, the alterations in the quality of the two murmurs, have a diagnostic value much greater than the modifications of the inspiratory murmur, and than the dry, hard, and difficult character of the two murmurs. The changes of quality form a continued chain, which embraces in its extent the most considerable portion of pulmonary phthisis. The hard, rough, dry character belongs more especially to the first period of phthisis; for it ceases to be appreciable as soon as the changes of quality have affected the respiratory sounds.

Such then is the aggregate of the signs furnished by auscultation of the respiration in the first stage of phthisis. We find that our analysis has now run to such a length that we must defer our further notice of this interesting work to some future time. Before, however, we close our present notice, we beg leave to correct an erroneous impression which M. Fournet seems to lie under; he seems to think that he was the first person who distinguished the respiratory murmur into the inspiratory and expiratory. Not to mention several physicians of our acquaintance, we beg to refer the reader to Schill's Semeiology,* § 332, an analysis of which appeared a long time since in this Journal: where he says, "In the normal state the sound of inspiration exceeds that of expiration as well in duration, as distinctness and audibility.—A deviation from these relations is a sign of disease."

* Translated by Spillan, London, 1839.

